# Association Between the Dietary Index for Gut Microbiota and Frailty: Mediating Role of Body Mass Index

**DOI:** 10.1002/fsn3.70607

**Published:** 2025-07-14

**Authors:** Chenyu Jiang, Luqi Zhu, Xiaosheng Teng, Gang Lin, Hongxun Wen, Zhenjun Yu, Shaojie Duan, Wenyuan Yang, Yaojian Shao

**Affiliations:** ^1^ Department of Geriatric Taizhou Central Hospital (Taizhou University Hospital) Zhejiang China; ^2^ Department of Critical Care Medicine Taizhou Hospital of Zhejiang Province Affiliated to Wenzhou Medical University Zhejiang China; ^3^ Department of Gastroenterology Taizhou Central Hospital (Taizhou University Hospital) Zhejiang China

**Keywords:** body mass index, diet, dietary index for gut microbiota, frailty, mediation, NHANES

## Abstract

Dietary composition and quality have been linked to the onset of frailty. Although the recently developed Dietary Index for Gut Microbiota (DI‐GM) offers a means to evaluate diet quality based on its impact on the gut microbiota, its relevance to frailty has yet to be determined. This cross‐sectional study utilized data from the NHANES collected between 2007 and 2020. DI‐GM scores were determined based on 14 dietary components, with higher scores indicating a diet more supportive of gut microbiota health. Frailty was evaluated using a validated 49‐item frailty index, with frailty defined as a score of 0.21 or higher. Weighted logistic regression, restricted cubic spline models, and mediation analyses were employed to examine the relationship between DI‐GM scores and frailty. A total of 27,026 eligible participants were included in the analysis. Higher DI‐GM scores were significantly associated with a lower risk of frailty. After full adjustment (Model 3), each one‐point increase in the DI‐GM scores corresponded to a 4% reduction in the odds of frailty (OR: 0.96, 95% CI: 0.92–1.00). Compared to the lowest quartile (Q1), individuals in the highest quartile (Q4) demonstrated significantly lower odds of frailty (OR: 0.81, 95% CI: 0.67–0.99). A nonlinear relationship between DI‐GM scores and frailty was identified (*p* for nonlinearity = 0.031). Age, sex, education level, and smoking status were found to potentially moderate this association. Mediation analyses further revealed that body mass index (BMI) partially mediated the relationship, accounting for 24.83% of the effect (*p* < 0.001). These results indicate that higher DI‐GM scores are linked to a lower risk of frailty, with BMI partially mediating this relationship. Future longitudinal studies are needed to establish causality and further investigate the underlying mechanisms.

## Graphical abstract

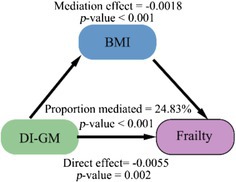

## 1 Introduction

Frailty is a complex syndrome observed in older adults, marked by reduced physiological reserves and an increased vulnerability to adverse health outcomes such as disability, hospitalization, and mortality (Angulo et al. 2016; Clegg et al. 2013; The Lancet 2019). With the aging of global populations, frailty is poised to emerge as a significant public health challenge (Hoogendijk et al. 2019). Because frailty is dynamic and potentially reversible, early identification and timely intervention are essential for delaying or reversing its progression (Lee et al. 2014).

Recent evidence underscores the pivotal role of dietary factors in influencing frailty risk, potentially through their impact on the composition and function of the gut microbiota (Cui et al. 2023; Li et al. 2025; O'Toole 2024). The gut microbiota, a complex and diverse microbial ecosystem, is increasingly recognized as a vital contributor to human health, playing key roles in digestion, nutrient absorption, immune function, and metabolic homeostasis (Jandhyala et al. 2015; Li, Xiong, et al. 2024). Dysbiosis, or an imbalance in microbial communities, has been linked to intestinal barrier dysfunction, increased inflammatory responses, and metabolic disturbances, all of which play a role in the development of frailty (Reid et al. 2011). Dietary patterns significantly shape gut microbiota diversity and metabolic outputs, affecting host metabolic health (Illiano et al. 2020). Research studies have demonstrated that higher intake of foods containing live microbes, such as fermented foods or unpeeled fruits and vegetables, is associated with a reduced risk of frailty (Dehghan et al. 2018; Ghoreishy et al. 2021). Furthermore, following dietary patterns such as the Mediterranean diet, anti‐inflammatory diet, and diets with high composite dietary antioxidant indices has been associated with a lower prevalence of frailty (Gross et al. 2025; Jung et al. 2024; Li, Wang, et al. 2024).

Although interest in the relationship between diet, gut microbiota, and overall health is increasing, there has been limited research on the interaction between diet and gut microbiota. Kase et al. conducted a systematic review of 106 articles to address this gap. They identified 14 dietary components that influence the gut microbiota in various ways, ultimately leading to the creation and formal designation of the Dietary Index for Gut Microbiota (DI‐GM) (Kase et al. 2024). The DI‐GM provides a standardized approach for evaluating dietary quality concerning gut microbiota health, promoting interdisciplinary research between nutrition and microbiology. However, the connection between the DI‐GM and frailty remains largely unexplored.

Given the modifiable nature of diet and its potential influence on gut microbiota composition, investigating the link between DI‐GM and frailty could offer valuable insights for developing dietary strategies to reduce frailty risk. This study was designed to closely examine the interaction between DI‐GM and frailty using data from NHANES, a large, nationally representative dataset. By exploring this relationship, the study aims to improve understanding of the diet‐gut microbiota‐frailty connection and inform future dietary recommendations for frailty prevention.

## 2 Methods

### 2.1 Data Collection

**FIGURE 1:**
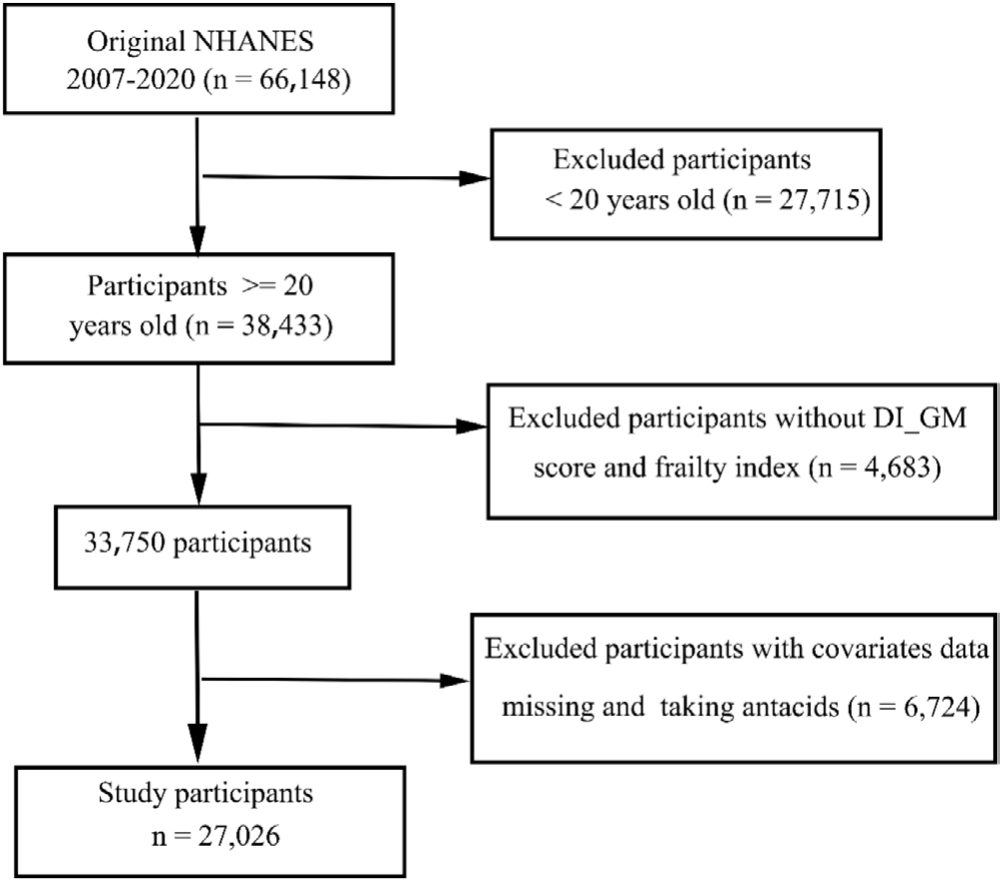
Participants flow chart.

This study used data from NHANES, which was designed to assess the health and nutritional status of Americans through a nationally representative survey employing complex, multistage probability sampling. Data were collected from seven consecutive NHANES cycles between 2007 and 2020. The initial dataset included 66,148 participants. Exclusion criteria included individuals under 20 years of age (*n* = 27,715), those with missing DI‐GM data or unreliable frailty index assessments (*n* = 4683), and participants with missing covariate information (*n* = 6700) or those using antacids (*n* = 24). After applying these exclusions, the final analytical sample consisted of 27,026 participants (Figure 1).

### 2.2 DI‐GM Calculation

DI‐GM scores were calculated following the scoring criteria established by Kase et al. The index consists of 14 dietary components categorized as beneficial or unfavorable for gut microbiota health (Kase et al. 2024). Beneficial dietary components included cranberries, avocado, fiber, broccoli, chickpeas, coffee, soybeans, fermented dairy, and whole grains. Due to limitations in NHANES dietary data, green tea was excluded from the analysis. Unfavorable components consisted of processed meat, red meat, refined cereals, and diets with fat making up 40% or more of total energy intake. DI‐GM scores were calculated based on 24‐h food recall data from NHANES cycles (2007–2020). Individuals whose consumption exceeded the sex‐specific median for each beneficial food component received a score of 1, while those below the median scored 0. In comparison, consumption above the median received a score of 0 for unfavorable components, and below the median received a score of 1. Total scores ranged from 0 to 14, with higher scores reflecting more gut‐friendly diets. Participants were grouped into quartiles based on their total DI‐GM scores: 0–4 (Q1), 5 (Q2), 6 (Q3), and ≥ 7 (Q4).

### 2.3 Frailty Index Calculations

Frailty was assessed using the validated frailty index developed by Wael Sabbah et al., following the standard procedures established by Searle and colleagues. This index includes 49 diagnostic criteria across seven domains: cognition (1 item), dependency (16 items), depression (7 items), comorbidities (13 items), hospitalization/nursing home use (5 items), anthropometrics (1 item), and laboratory markers (6 items). Each item was scored from 0 (no deficit) to 1 (most severe deficit), and the frailty index was calculated by dividing the total score by the number of completed items. Frailty was defined as a frailty index score of ≥ 0.21 (Sun et al. 2024). For further details regarding scoring, see Table S1.

### 2.4 Study Covariates

Potential confounding factors influencing frailty were carefully considered based on the references (Huo et al. 2024; Jia et al. 2024). Additional participant data extracted from the NHANES database for this study included ethnicity (Mexican American, Non‐Hispanic White, Other Hispanic, Non‐Hispanic Black, and other), age (in years), sex (male or female), family poverty‐to‐income ratio (PIR), education level (high schoo or below, and above high school), water intake (total tap and bottled water intake, in grams), energy intake (in kcal), BMI (kg/m^2^), physical activity (including recreational and work activities), smoking status (non‐smoker [< 100 lifetime cigarettes or > 100 lifetime cigarettes but not a current smoker], smoker [> 100 lifetime cigarettes and currently smoking]), alcohol consumption (non‐drinker [< 12 lifetime drinks or 12+ per year but none in the past year], drinker [within the previous 12 months]), as well as the presence of hypertension, diabetes mellitus (DM), and cardiovascular disease (CVD). The DM was defined by self‐reported diagnosis, use of insulin or antidiabetic medications, fasting blood glucose (FBG) ≥ 126 mg/dL, HbA1c ≥ 6.5%, or serum glucose ≥ 200 mg/dL 2 h post‐75 g oral glucose challenge. Hypertension was defined as an average systolic blood pressure ≥ 140 mmHg and/or diastolic blood pressure ≥ 90 mmHg across three consecutive measurements, a history of antihypertensive treatment, or a self‐reported diagnosis of hypertension. CVD was defined by self‐reported history of coronary heart disease, heart attack, or stroke.

### 2.5 Statistical Analyses

Sample weights were applied to all analyses to adjust for the multistage sampling design of NHANES. Categorical variables are presented as counts (weighted percentages) and analyzed using chi‐squared tests, while continuous variables are reported as means ± standard error (SE) and examined using Student's *t*‐tests. Weighted univariate and multivariate logistic regression models were employed to explore associations between DI‐GM scores and frailty, with odds ratios (ORs) and 95% confidence intervals (CIs) generated. Model 1 was unadjusted, Model 2 adjusted for age, sex, education, and ethnicity, and Model 3 included additional adjustments for BMI, physical activity, energy and water intake, smoking status, alcohol consumption, DM, hypertension, and CVD. Nonlinear associations were assessed using restricted cubic spline (RCS) models. Subgroup analyses were performed based on age (< 60, ≥ 60), sex, ethnicity, education, BMI (< 25, 25–30, > 30 kg/m^2^), physical activity, DM, hypertension, CVD, smoking, and alcohol consumption. Mediation analysis and stratified mediation analysis were conducted using the “mediation” package in R with 1000 bootstrap iterations to assess the mediating role of BMI in the DI‐GM‐frailty relationship. All analyses were performed in R (v 4.2.2), with *p* ≤ 0.05 considered statistically significant.

## 3 Results

### 3.1 Participants

A total of 27,026 participants were included in this study and categorized into quartiles based on their DI‐GM scores. Compared to those in the lowest quartile (Q1), a smaller proportion of participants in the highest quartile (Q4) were classified as frail. Furthermore, higher DI‐GM scores were associated with a greater proportion of females, non‐Hispanic Whites, individuals with higher educational attainment, and those with a higher PIR. Participants in Q4 also reported higher water intake, lower BMI, a reduced prevalence of DM, fewer smokers, and a higher proportion of alcohol consumers compared to those in Q1. A summary of baseline characteristics is presented in Table 1.

**TABLE 1:** Basic participant characteristics classified according to their DI‐GM scores.

	DI‐GM				*p*
	Q1 [0–4]	Q2 [5]	Q3 [6]	Q4 [7–11]	
Age (years)	45.64 (0.28)	47.65 (0.37)	49.31 (0.41)	50.67 (0.51)	< 0.0001
Sex (%)					
Female	6435 (47.91)	3145 (51.60)	2181 (51.90)	1822 (58.28)	< 0.0001
Male	6860 (52.09)	3108 (48.40)	2029 (48.10)	1446 (41.72)	
Ethnicity (%)					
Mexican American	1952 (9.16)	950 (8.57)	558 (6.74)	348 (4.82)	< 0.0001
Non‐Hispanic Black	3366 (13.47)	1284 (9.94)	742 (7.87)	438 (5.75)	
Non‐Hispanic White	5296 (64.18)	2741 (69.53)	2001 (72.09)	1706 (76.62)	
Other Hispanic	1409 (6.41)	601 (5.01)	398 (4.85)	287 (4.08)	
Other Race	1272 (6.78)	677 (6.94)	511 (8.45)	489 (8.73)	
Education (%)					
Less than high school	1290 (5.40)	569 (4.51)	327 (3.58)	173 (2.10)	< 0.0001
High school	5443 (38.53)	2176 (31.89)	1298 (26.97)	846 (22.46)	
More than high school	6562 (56.07)	3508 (63.60)	2585 (69.45)	2249 (75.44)	
PIR	2.78 (0.04)	3.09 (0.05)	3.23 (0.05)	3.47 (0.04)	< 0.0001
Water intake (g)	1080.76 (16.86)	1087.13 (24.06)	1147.68 (27.73)	1226.18 (30.29)	< 0.0001
Energy intake (kcal)	2082.79 (11.72)	2061.34 (17.66)	2117.48 (18.27)	2108.10 (15.90)	0.05
Body mass index (BMI, kg/m^2^)	29.71 (0.11)	28.98 (0.12)	28.63 (0.16)	28.05 (0.18)	< 0.0001
< 25	3497 (26.42)	1742 (29.56)	1232 (30.91)	1098 (36.33)	
25–30	4274 (32.59)	2066 (33.52)	1429 (34.41)	1132 (33.39)	
> 30	5524 (40.99)	2445 (36.92)	1549 (34.68)	1038 (30.28)	
Physical activity (%)					
No	4500 (27.98)	1943 (25.69)	1239 (23.94)	825 (20.72)	< 0.0001
Yes	8795 (72.02)	4310 (74.31)	2971 (76.06)	2443 (79.28)	
DM (%)					
No	10645 (84.54)	5138 (86.44)	3449 (87.12)	2712 (86.66)	0.003
Yes	2650 (15.46)	1115 (13.56)	761 (12.88)	556 (13.34)	
Hypertension (%)					
No	7534 (61.49)	3583 (61.99)	2440 (62.34)	1876 (63.38)	0.53
Yes	5761 (38.51)	2670 (38.01)	1770 (37.66)	1392 (36.62)	
CVD (%)					
No	11815 (91.28)	5571 (91.07)	3776 (91.58)	2931 (91.48)	0.89
Yes	1480 (8.72)	682 (8.93)	434 (8.42)	337 (8.52)	
Smoke (%)					
No	10207 (77.37)	4969 (80.05)	3485 (82.38)	2822 (86.14)	< 0.0001
Yes	3088 (22.63)	1284 (19.95)	725 (17.62)	446 (13.86)	
Alcohol user (%)					
No	3737 (23.83)	1744 (23.01)	1169 (21.56)	879 (21.30)	0.04
Yes	9558 (76.17)	4509 (76.99)	3041 (78.44)	2389 (78.70)	
Frailty (%)					
No	10086 (80.85)	4856 (81.62)	3315 (83.86)	2654 (84.81)	< 0.0001
Yes	3209 (19.15)	1397 (18.38)	895 (16.14)	614 (15.19)	

Abbreviations: BMI, body mass index; CVD, cardiovascular disease; DI‐GM, dietary index for gut microbiota; DM, diabetes mellitus; PIR, poverty income ratio.

### 3.2 Association of the DI‐GM and Frailty

**FIGURE 2:**
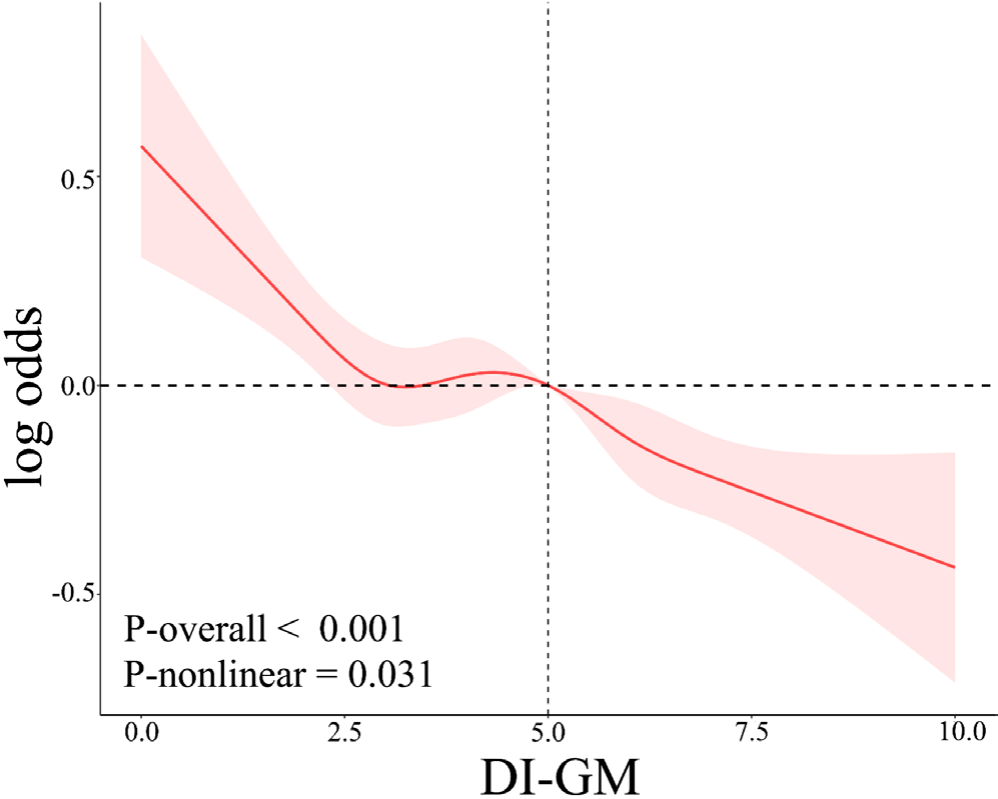
RCS curves corresponding to the association between DI‐GM scores and frailty.

Weighted logistic regression analyses were conducted to examine the associations between DI‐GM scores and frailty (Table 2). In the unadjusted Model 1, higher DI‐GM scores were associated with a decreased likelihood of frailty (OR: 0.93, 95% CI: 0.91–0.96). Compared to Q1, participants in Q3 (OR: 0.81, 95% CI: 0.72–0.92) and Q4 (OR: 0.76, 95% CI: 0.65–0.88) demonstrated significantly lower odds of frailty. After adjusting for age, sex, education, and ethnicity in Model 2, the inverse association between DI‐GM score and frailty remained significant (OR: 0.91, 95% CI: 0.88–0.94). When compared to Q1, the odds of frailty were substantially lower in Q3 (OR: 0.75, 95% CI: 0.66–0.86) and Q4 (OR: 0.68, 95% CI: 0.58–0.79), representing reductions of 25% and 32%, respectively. In the fully adjusted Model 3, each one‐point increase in DI‐GM score was linked to a 4% reduction in the likelihood of frailty (OR: 0.96, 95% CI: 0.92–1.00). Compared to Q1, significantly lower odds of frailty were observed only in Q4 (OR: 0.81, 95% CI: 0.67–0.99). The overall trend analysis revealed a *p* value of 0.02. The RCS models were employed to explore potential nonlinearity. As shown in Figure 2, the odds of frailty decreased as DI‐GM scores increased (*p* < 0.001), with a significant nonlinear association observed (*p* for nonlinearity = 0.031).

**TABLE 2:** Relationships between DI‐GM scores and participant frailty.

	Model 1		Model 2		Model 3	
	OR (95% CI)	*p*	OR (95% CI)	*p*	OR (95% CI)	*p*
DI‐GM	0.93 (0.91, 0.96)	< 0.0001	0.91 (0.88, 0.94)	< 0.0001	0.96 (0.92, 1.00)	0.05
DI‐GM quartile						
Q1 [0–4]	ref		ref		ref	
Q2 [5]	0.95 (0.86, 1.05)	0.29	0.92 (0.83, 1.02)	0.11	1.03 (0.92, 1.15)	0.58
Q3 [6]	0.81 (0.72, 0.92)	0.001	0.75 (0.66, 0.86)	< 0.0001	0.89 (0.77, 1.03)	0.11
Q4 [7–11]	0.76 (0.65, 0.88)	< 0.001	0.68 (0.58, 0.79)	< 0.0001	0.81 (0.67, 0.99)	0.04
*p* for trend		< 0.0001		< 0.0001		0.02

*Note:* Model 1 did not adjust for covariates; Model 2 adjusted for age, sex, education, and ethnicity; Model 3 adjusted for age, sex, education, ethnicity, BMI, energy and water intake, smoking status, alcohol consumption, DM, hypertension, and CVD.

Abbreviations: CI, confidence interval; DI‐GM, dietary index for gut microbiota; OR, odds ratio.

### 3.3 Subgroup Analysis

**FIGURE 3:**
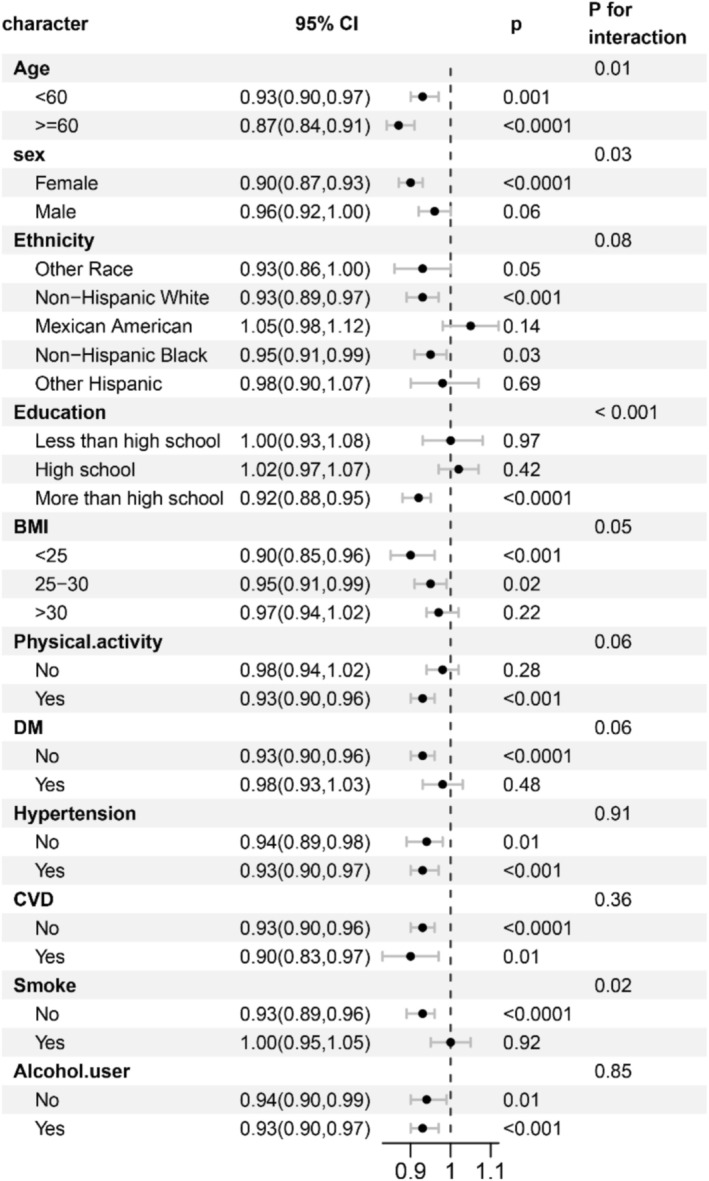
Subgroup analyses corresponding to the association between DI‐GM and frailty.

Stratified analyses were conducted across various subgroups. As shown in Figure 3, the inverse association between DI‐GM scores and frailty was evident in the following subgroups: age (< 60, ≥ 60), sex (female), ethnicity (Non‐Hispanic White, Non‐Hispanic Black, Other), education level (> high school), BMI (< 25, 25–30 kg/m^2^), and physical activity. This relationship was also significant among participants without DM, hypertension, or CVD, as well as among non‐smokers and alcohol consumers. Significant interactions were observed between DI‐GM scores and age (*p* for interaction = 0.01), sex (*p* for interaction = 0.03), education (*p* for interaction < 0.001), and smoking status (*p* for interaction = 0.02) regarding frailty risk.

### 3.4 Mediation Analyses

**FIGURE 4:**
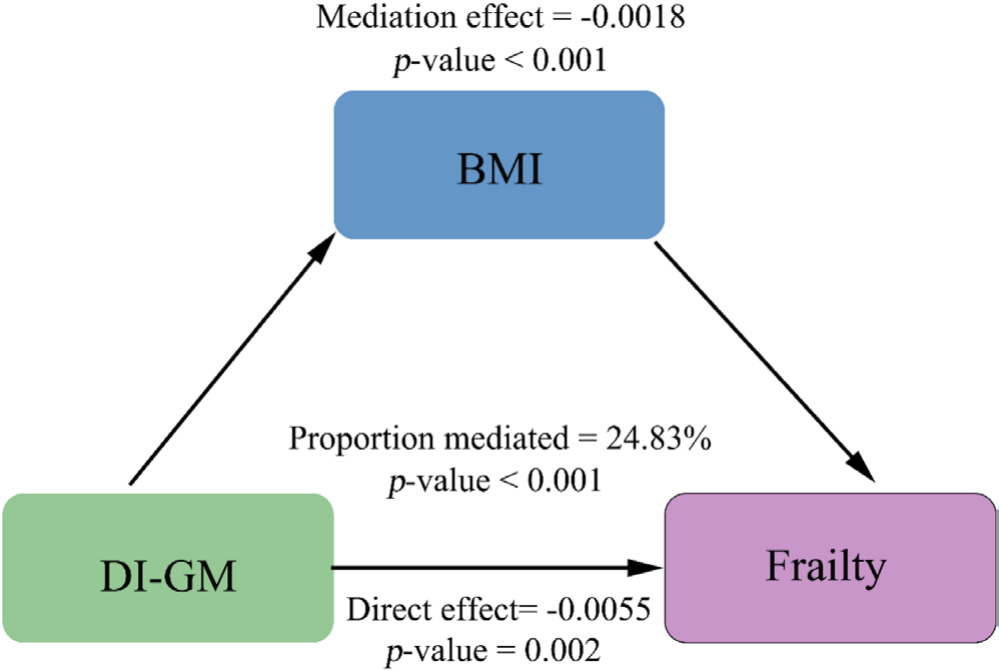
The mediating role of BMI in the association between DI‐GM and frailty.

Mediation analyses were conducted to investigate further the mechanisms underlying the relationship between DI‐GM and frailty. A significant mediating effect was found for BMI (Figure 4). The overall effect coefficient was −0.0073 (*p* < 0.001), with a mediating effect of −0.0018 (*p* < 0.001), accounting for 24.83% of the total effect (*p* < 0.001). Stratified mediation analyses revealed substantial heterogeneity; BMI mediation was observed exclusively among individuals with obesity (BMI > 30 kg/m^2^), with no significant mediation detected in those with lower BMI categories (Figure S1).

## 4 Discussion

This extensive observational study of American adults revealed a significant inverse association between DI‐GM scores and frailty. Further RCS analysis indicated a potential nonlinear relationship between these variables. Furthermore, age, sex, education, and smoking status were identified as potential moderators of this relationship. Mediation analysis further showed that BMI played a significant role in mediating the DI‐GM–frailty connection.

A healthy and varied gut microbiome is crucial for preserving intestinal barrier function, immune health, metabolic stability, and overall physiological resilience, all of which play a role in preventing frailty. Recent findings indicate that the gut microbiota affects systemic health by releasing bioactive compounds into the bloodstream, which then influence various physiological processes (Chen et al. 2021; Gagnon et al. 2023). A meta‐analysis of 11 studies highlighted substantial alterations in over 50 microbial species among frail individuals (Almeida et al. 2022). Importantly, frailty has been associated with an increased abundance of pro‐inflammatory microbes, such as Akkermansia, and a reduction in beneficial, anti‐inflammatory bacteria (Xu et al. 2021). Since diet plays a crucial role in shaping the composition of the gut microbiota, dietary changes could be a promising approach to improve gut health and reduce frailty risk. Mediterranean diets, rich in fruits, healthy fats, whole grains, and vegetables, have been associated with lower systemic inflammation, improved muscle strength, and a reduced prevalence of frailty (Gross et al. 2025). A study by Tarini Shankar Ghosh showed that following a Mediterranean‐style diet reduced frailty in older adults by altering gut microbiota composition, increasing levels of both short‐ and branched‐chain fatty acids, decreasing secondary bile acids, and lowering inflammatory markers such as IL‐17 and C‐reactive protein, among other factors (Ghosh et al. 2020). Moreover, increased consumption of non‐starchy vegetables and fruits has been linked to a reduced frailty risk.

In a systematic review of 106 articles, Kase et al. (2024) identified 14 food groups and nutrients linked to gut microbiota, forming the DI‐GM foundation. These findings offer new insights, showing that higher DI‐GM scores are strongly linked to lower levels of frailty. Diets high in fiber, fermented foods, and plant‐based nutrients are essential elements of the DI‐GM, promote the growth of beneficial microorganisms like *Bifidobacterium* and *Lactobacillus* (Li et al. 2020; Ruiz‐Saavedra et al. 2023). These bacteria generate short‐chain fatty acids (SCFAs), which improve gut barrier function and lower systemic inflammation (Parada Venegas et al. 2019). In comparison, diets rich in red meat, processed foods, and saturated fats—components deemed harmful in the DI‐GM are—linked to gut dysbiosis, marked by an overgrowth of harmful bacteria and increased intestinal permeability. This imbalance may contribute to chronic inflammation and frailty. Furthermore, recent studies have shown the health benefits of probiotics (Wang et al. 2023; Yu et al. 2021). For example, supplementation with *Lactobacillus paracasei* PS23 has been demonstrated to modify gut microbiota, reduce oxidative stress and inflammation, and improve cognitive function in aging mice (Cheng et al. 2022). Furthermore, *Lactobacillus acidophilus* and *Bifidobacterium lactis* supplementation has been associated with increased erythrocyte antioxidant enzyme activity (Ejtahed et al. 2011). Aimée Parker's study further showed that fecal microbiota transplantation from young to aged mice effectively lowered pro‐inflammatory cytokine levels, boosting microbial diversity and increasing beneficial metabolites like acetate and tauro‐conjugated bile acids (Parker et al. 2022). Subgroup analyses revealed statistically significant interactions between DI‐GM scores and age, sex, education, and smoking status. The inverse relationship between DI‐GM and frailty was more pronounced in older adults (Collard et al. 2012), females (Kane and Howlett 2021), lower educational status (Bellelli et al. 2023) and non‐smokers. Socioeconomic factors linked to education may help explain this interaction, as higher educational attainment often leads to better access to nutrient‐rich diets due to increased financial resources and health literacy, thus supporting healthier lifestyle choices (Hanlon et al. 2024; Wennberg et al. 2023). This association was absent among smokers, potentially due to the detrimental effects of smoking on oxidative stress, inflammation, and organ function (Rahman and Laher 2007). Smoking has also been shown to impair nutrient absorption, including vitamin C, therefore diminishing the nutritional benefits of a gut‐friendly diet (Wilson et al. 2017; Yoshihara et al. 2022). Taken together, these findings suggest that smoking may reduce the protective benefits of a microbiota‐supportive diet in preventing frailty.

A major finding of this study was that BMI mediates the relationship between DI‐GM and frailty, with stratified analyses indicating that this mediation was most pronounced in individuals with obesity (BMI > 30 kg/m^2^). A study by Kulapong Jayanama also showed a significant increase in frailty index scores as BMI rose in middle‐aged and older adults (Jayanama et al. 2022). The link between obesity and frailty may be explained by metabolic and inflammatory pathways (Jayanama et al. 2022). Poor dietary habits and gut microbiota dysbiosis have been implicated in obesity, affecting energy balance and fat storage, thus contributing to metabolic syndrome (Musso et al. 2010). A randomized controlled trial involving premenopausal overweight women found that consuming fermented dairy products significantly reduced blood lipid levels compared to consuming low‐fat milk (Fathi et al. 2017). Furthermore, obesity triggers chronic low‐grade inflammation, with adipose tissue producing pro‐inflammatory mediators such as TNF‐α and IL‐6 (Simoes et al. 2021). Persistent inflammation may impair cellular function and accelerate aging, increasing frailty risk (Walker et al. 2022). These findings indicate that dietary modulation of the gut microbiota may reduce frailty risk through two primary mechanisms: lowering systemic inflammation and regulating body weight. Importantly, this protective effect seems especially evident among individuals with obesity, where gut microbiota‐host metabolic interactions predominantly mediate the link between diet and frailty through adiposity‐related pathways.

Given the potential for bidirectional relationships, the observed associations between DI‐GM scores and frailty should be interpreted with caution. The cross‐sectional design precludes ruling out reverse causality, whereby existing frailty may influence dietary choices and intake through mechanisms such as sensory deficits, physical limitations, and inflammation‐related appetite changes (Kuczmarski et al. 2023; Moradell et al. 2021; Tanaka et al. 2024). These factors may contribute to a bidirectional relationship, wherein frailty leads to dietary alterations that, in turn, exacerbate microbial dysbiosis forming a “frailty‐diet‐microbiota vicious cycle” that warrants further investigation through longitudinal research.

This study has several significant strengths, including the use of a large, nationally representative cohort and the application of comprehensive statistical analyses, both of which improve the robustness and generalizability of the findings. However, certain limitations must be acknowledged. First, the cross‐sectional design precludes establishing causal relationships, highlighting the need for longitudinal studies and randomized controlled trials to clarify the directionality of these associations. Specifically, we cannot rule out the possibility of reverse causation, whereby pre‐existing frailty might drive alterations in dietary patterns. Second, dietary information was collected through 24‐h recalls, subject to recall bias. Third, long‐term nutritional habits and seasonal variations in food intake were not captured. Lastly, despite adjusting for multiple covariates, the possibility of residual confounding cannot be excluded. Future research should prioritize longitudinal designs and include more diverse populations to elucidate better the mechanistic pathways linking diet, gut microbiota, and frailty. Moreover, personalized dietary strategies tailored to individual variability and specific demographic groups should be explored to advance targeted nutritional interventions to promote healthy aging and reduce frailty.

## 5 Conclusions

In summary, this study demonstrates that higher DI‐GM scores are associated with a lower risk of frailty, with BMI partially mediating this relationship (accounting for 24.83% of the effect). Future longitudinal studies are essential to confirm causality, elucidate the underlying mechanisms, and validate these associations in diverse populations. These findings offer a valuable framework for designing targeted dietary strategies and personalized interventions to foster healthy aging and mitigate frailty risk.

## Author Contributions

All authors contributed to the study conception and design. Y.S., C.J., and L.Z.: conceptualization, data curation, formal analysis, investigation, methodology, software, validation, visualization, writing – original draft, writing – review and editing. H.W., X.T., G.L., and W.Y.: conceptualization, writing – review and editing. Z.Y. and S.D.: conceptualization, data curation, formal analysis, investigation, methodology, writing – review and editing. All authors contributed to the article and approved the submitted version.

## Ethics Statement

The studies involving human participants were reviewed and approved by the National Center for Health Statistics Research Ethics Review Board. All methods were carried out in accordance with relevant guidelines and regulations (Declaration of Helsinki).

## Consent

The authors have nothing to report.

## Conflicts of Interest

The authors declare no conflicts of interest.

## Supporting information


Data S1.

## Data Availability

The data used in this study are publicly available from the National Health and Nutrition Examination Survey (NHANES) database, which is maintained by the Centers for Disease Control and Prevention (CDC). All datasets utilized in this manuscript can be accessed at NHANES website link: https://www.cdc.gov/nchs/nhanes/. The specific variables and analytical methods used in this study are described in detail in the Methods section of the manuscript. Researchers interested in replicating or building upon our findings can access the data freely and follow the same protocols outlined in our study.

## References

[fsn370607-bib-0001] Almeida, H. M. , A. V. Sardeli , J. Conway , N. A. Duggal , and C. R. Cavaglieri . 2022. “Comparison Between Frail and Non‐Frail Older Adults' Gut Microbiota: A Systematic Review and Meta‐Analysis.” Ageing Research Reviews 82: 101773. 10.1016/j.arr.2022.101773. 36349647

[fsn370607-bib-0002] Angulo, J. , M. El Assar , and L. Rodríguez‐Mañas . 2016. “Frailty and Sarcopenia as the Basis for the Phenotypic Manifestation of Chronic Diseases in Older Adults.” Molecular Aspects of Medicine 50: 1–32. 10.1016/j.mam.2016.06.001. 27370407

[fsn370607-bib-0003] Bellelli, F. , E. Consorti , T. M. K. Hettiarachchige , et al. 2023. “Relationship Among Age, Education and Frailty in Older Persons.” Journal of Frailty & Aging 12, no. 4: 326–328. 10.14283/jfa.2023.39. 38008985

[fsn370607-bib-0004] Chen, Y. , J. Zhou , and L. Wang . 2021. “Role and Mechanism of Gut Microbiota in Human Disease.” Frontiers in Cellular and Infection Microbiology 11: 625913. 10.3389/fcimb.2021.625913. 33816335 PMC8010197

[fsn370607-bib-0005] Cheng, L. H. , P. Y. Chou , A. T. Hou , C. L. Huang , W. L. Shiu , and S. Wang . 2022. “ *Lactobacillus paracasei* PS23 Improves Cognitive Deficits via Modulating the Hippocampal Gene Expression and the Gut Microbiota in D‐Galactose‐Induced Aging Mice.” Food & Function 13, no. 9: 5240–5251. 10.1039/d2fo00165a. 35438699

[fsn370607-bib-0006] Clegg, A. , J. Young , S. Iliffe , M. O. Rikkert , and K. Rockwood . 2013. “Frailty in Elderly People.” Lancet 381, no. 9868: 752–762. 10.1016/s0140-6736(12)62167-9. 23395245 PMC4098658

[fsn370607-bib-0007] Collard, R. M. , H. Boter , R. A. Schoevers , and R. C. Oude Voshaar . 2012. “Prevalence of Frailty in Community‐Dwelling Older Persons: A Systematic Review.” Journal of the American Geriatrics Society 60, no. 8: 1487–1492. 10.1111/j.1532-5415.2012.04054.x. 22881367

[fsn370607-bib-0008] Cui, G. , S. Li , H. Ye , et al. 2023. “Gut Microbiome and Frailty: Insight From Genetic Correlation and Mendelian Randomization.” Gut Microbes 15, no. 2: 2282795. 10.1080/19490976.2023.2282795. 37990415 PMC10730212

[fsn370607-bib-0009] Dehghan, M. , A. Mente , S. Rangarajan , et al. 2018. “Association of Dairy Intake With Cardiovascular Disease and Mortality in 21 Countries From Five Continents (PURE): A Prospective Cohort Study.” Lancet 392, no. 10161: 2288–2297. 10.1016/s0140-6736(18)31812-9. 30217460

[fsn370607-bib-0010] Ejtahed, H. S. , J. Mohtadi‐Nia , A. Homayouni‐Rad , et al. 2011. “Effect of Probiotic Yogurt Containing Lactobacillus Acidophilus and *Bifidobacterium lactis* on Lipid Profile in Individuals With Type 2 Diabetes Mellitus.” Journal of Dairy Science 94, no. 7: 3288–3294. 10.3168/jds.2010-4128. 21700013

[fsn370607-bib-0011] Fathi, Y. , N. Ghodrati , M. J. Zibaeenezhad , and S. Faghih . 2017. “Kefir Drink Causes a Significant Yet Similar Improvement in Serum Lipid Profile, Compared With Low‐Fat Milk, in a Dairy‐Rich Diet in Overweight or Obese Premenopausal Women: A Randomized Controlled Trial.” Journal of Clinical Lipidology 11, no. 1: 136–146. 10.1016/j.jacl.2016.10.016. 28391880

[fsn370607-bib-0012] Gagnon, E. , P. L. Mitchell , H. D. Manikpurage , et al. 2023. “Impact of the Gut Microbiota and Associated Metabolites on Cardiometabolic Traits, Chronic Diseases and Human Longevity: A Mendelian Randomization Study.” Journal of Translational Medicine 21, no. 1: 60. 10.1186/s12967-022-03799-5. 36717893 PMC9887809

[fsn370607-bib-0013] Ghoreishy, S. M. , F. Asoudeh , A. Jayedi , and H. Mohammadi . 2021. “Fruit and Vegetable Intake and Risk of Frailty: A Systematic Review and Dose Response Meta‐Analysis.” Ageing Research Reviews 71: 101460. 10.1016/j.arr.2021.101460. 34534684

[fsn370607-bib-0014] Ghosh, T. S. , S. Rampelli , I. B. Jeffery , et al. 2020. “Mediterranean Diet Intervention Alters the Gut Microbiome in Older People Reducing Frailty and Improving Health Status: The NU‐AGE 1‐Year Dietary Intervention Across Five European Countries.” Gut 69, no. 7: 1218–1228. 10.1136/gutjnl-2019-319654. 32066625 PMC7306987

[fsn370607-bib-0015] Gross, D. C. , J. C. Dahringer , P. Bramblett , et al. 2025. “The Relationship Between a Mediterranean Diet and Frailty in Older Adults: NHANES 2007–2017.” Nutrients 17, no. 2: 326. 10.3390/nu17020326. 39861456 PMC11767853

[fsn370607-bib-0016] Hanlon, P. , M. Politis , H. Wightman , et al. 2024. “Frailty and Socioeconomic Position: A Systematic Review of Observational Studies.” Ageing Research Reviews 100: 102420. 10.1016/j.arr.2024.102420. 39025269

[fsn370607-bib-0017] Hoogendijk, E. O. , J. Afilalo , K. E. Ensrud , P. Kowal , G. Onder , and L. P. Fried . 2019. “Frailty: Implications for Clinical Practice and Public Health.” Lancet 394, no. 10206: 1365–1375. 10.1016/s0140-6736(19)31786-6. 31609228

[fsn370607-bib-0018] Huo, X. , S. Jia , L. Sun , Y. Yao , H. Liao , and X. Chen . 2024. “Association of Dietary Live Microbe Intake With Frailty in US Adults: Evidence From NHANES.” Journal of Nutrition, Health & Aging 28, no. 3: 100171. 10.1016/j.jnha.2024.100171.38423889

[fsn370607-bib-0019] Illiano, P. , R. Brambilla , and C. Parolini . 2020. “The Mutual Interplay of Gut Microbiota, Diet and Human Disease.” FEBS Journal 287, no. 5: 833–855. 10.1111/febs.15217. 31955527

[fsn370607-bib-0020] Jandhyala, S. M. , R. Talukdar , C. Subramanyam , H. Vuyyuru , M. Sasikala , and D. Nageshwar Reddy . 2015. “Role of the Normal Gut Microbiota.” World Journal of Gastroenterology 21, no. 29: 8787–8803. 10.3748/wjg.v21.i29.8787. 26269668 PMC4528021

[fsn370607-bib-0021] Jayanama, K. , O. Theou , J. Godin , A. Mayo , L. Cahill , and K. Rockwood . 2022. “Relationship of Body Mass Index With Frailty and All‐Cause Mortality Among Middle‐Aged and Older Adults.” BMC Medicine 20, no. 1: 404. 10.1186/s12916-022-02596-7. 36280863 PMC9594976

[fsn370607-bib-0022] Jia, X. , C. Su , J. Zhang , et al. 2024. “Age and Gender Disparities in the Association of Long‐Term Dietary Choline and Choline Compound Intakes With Incident Cognitive Decline in Middle‐Aged and Older Chinese Adults: A Prospective Cohort Study.” Nutrients 16, no. 23: 4121. 10.3390/nu16234121. 39683516 PMC11644459

[fsn370607-bib-0023] Jung, S. , Y. Lee , K. Kim , and S. Park . 2024. “Association of the Dietary Inflammatory Index With Sarcopenic Obesity and Frailty in Older Adults.” BMC Geriatrics 24, no. 1: 654. 10.1186/s12877-024-05239-z. 39097690 PMC11297761

[fsn370607-bib-0024] Kane, A. E. , and S. E. Howlett . 2021. “Sex Differences in Frailty: Comparisons Between Humans and Preclinical Models.” Mechanisms of Ageing and Development 198: 111546. 10.1016/j.mad.2021.111546. 34324923

[fsn370607-bib-0025] Kase, B. E. , A. D. Liese , J. Zhang , E. A. Murphy , L. Zhao , and S. E. Steck . 2024. “The Development and Evaluation of a Literature‐Based Dietary Index for Gut Microbiota.” Nutrients 16, no. 7: 1045. 10.3390/nu16071045. 38613077 PMC11013161

[fsn370607-bib-0026] Kuczmarski, M. F. , M. A. Beydoun , M. F. Georgescu , et al. 2023. “Pro‐Inflammatory Diets Are Associated With Frailty in an Urban Middle‐Aged African American and White Cohort.” Nutrients 15, no. 21: 4598. 10.3390/nu15214598. 37960250 PMC10648548

[fsn370607-bib-0027] Lee, J. S. , T. W. Auyeung , J. Leung , T. Kwok , and J. Woo . 2014. “Transitions in Frailty States Among Community‐Living Older Adults and Their Associated Factors.” Journal of the American Medical Directors Association 15, no. 4: 281–286. 10.1016/j.jamda.2013.12.002. 24534517

[fsn370607-bib-0028] Li, X. , Q. Wang , T. Ma , et al. 2024. “Dietary Inflammatory Index, Dietary Total Antioxidant Capacity, and Frailty Among Older Chinese Adults.” Journal of Nutrition, Health & Aging 28, no. 4: 100168. 10.1016/j.jnha.2024.100168.38341967

[fsn370607-bib-0029] Li, Y. , Q. Gong , W. He , and J. Ke . 2025. “Dietary Intake of Live Microbes and Its Association With Frailty in Older Adults: A NHANES Analysis (1999–2018).” BMC Geriatrics 25, no. 1: 91. 10.1186/s12877-025-05725-y. 39934741 PMC11817259

[fsn370607-bib-0030] Li, Y. J. , X. Chen , T. K. Kwan , et al. 2020. “Dietary Fiber Protects Against Diabetic Nephropathy Through Short‐Chain Fatty Acid‐Mediated Activation of G Protein‐Coupled Receptors GPR43 and GPR109A.” Journal of the American Society of Nephrology 31, no. 6: 1267–1281. 10.1681/asn.2019101029. 32358041 PMC7269358

[fsn370607-bib-0031] Li, Z. , W. Xiong , Z. Liang , et al. 2024. “Critical Role of the Gut Microbiota in Immune Responses and Cancer Immunotherapy.” Journal of Hematology & Oncology 17, no. 1: 33. 10.1186/s13045-024-01541-w. 38745196 PMC11094969

[fsn370607-bib-0032] Moradell, A. , Á. I. Fernández‐García , D. Navarrete‐Villanueva , et al. 2021. “Functional Frailty, Dietary Intake, and Risk of Malnutrition. Are Nutrients Involved in Muscle Synthesis the Key for Frailty Prevention?” Nutrients 13, no. 4: 1231. 10.3390/nu13041231. 33917848 PMC8068284

[fsn370607-bib-0033] Musso, G. , R. Gambino , and M. Cassader . 2010. “Obesity, Diabetes, and Gut Microbiota: The Hygiene Hypothesis Expanded?” Diabetes Care 33, no. 10: 2277–2284. 10.2337/dc10-0556. 20876708 PMC2945175

[fsn370607-bib-0034] O'Toole, P. W. 2024. “Ageing, Microbes and Health.” Microbial Biotechnology 17, no. 5: e14477. 10.1111/1751-7915.14477. 38801344 PMC11129672

[fsn370607-bib-0035] Parada Venegas, D. , M. K. De la Fuente , G. Landskron , et al. 2019. “Short Chain Fatty Acids (SCFAs)‐Mediated Gut Epithelial and Immune Regulation and Its Relevance for Inflammatory Bowel Diseases.” Frontiers in Immunology 10: 277. 10.3389/fimmu.2019.00277. 30915065 PMC6421268

[fsn370607-bib-0036] Parker, A. , S. Romano , R. Ansorge , et al. 2022. “Fecal Microbiota Transfer Between Young and Aged Mice Reverses Hallmarks of the Aging Gut, Eye, and Brain.” Microbiome 10, no. 1: 68. 10.1186/s40168-022-01243-w. 35501923 PMC9063061

[fsn370607-bib-0037] Rahman, M. M. , and I. Laher . 2007. “Structural and Functional Alteration of Blood Vessels Caused by Cigarette Smoking: An Overview of Molecular Mechanisms.” Current Vascular Pharmacology 5, no. 4: 276–292. 10.2174/157016107782023406. 17979794

[fsn370607-bib-0038] Reid, G. , J. A. Younes , H. C. Van der Mei , G. B. Gloor , R. Knight , and H. J. Busscher . 2011. “Microbiota Restoration: Natural and Supplemented Recovery of Human Microbial Communities.” Nature Reviews. Microbiology 9, no. 1: 27–38. 10.1038/nrmicro2473. 21113182

[fsn370607-bib-0039] Ruiz‐Saavedra, S. , C. González Del Rey , A. Suárez , et al. 2023. “Associations of Dietary Factors and Xenobiotic Intake With Faecal Microbiota Composition According to the Presence of Intestinal Mucosa Damage.” Food & Function 14, no. 21: 9591–9605. 10.1039/d3fo01356a. 37740374

[fsn370607-bib-0040] Simoes, E. , J. Correia‐Lima , L. Sardas , et al. 2021. “Sex Dimorphism in Inflammatory Response to Obesity in Childhood.” International Journal of Obesity (London) 45, no. 4: 879–887. 10.1038/s41366-021-00753-1. PMC8005372 33526854

[fsn370607-bib-0041] Sun, L. , X. Huo , S. Jia , and X. Chen . 2024. “The Association Between Circadian Syndrome and Frailty in US Adults: A Cross‐Sectional Study of NHANES Data From 2007 to 2018.” Aging Clinical and Experimental Research 36, no. 1: 105. 10.1007/s40520-024-02745-3. 38713270 PMC11076391

[fsn370607-bib-0042] Tanaka, T. , H. Hirano , K. Ikebe , et al. 2024. “Consensus Statement on ‘Oral Frailty’ From the Japan Geriatrics Society, the Japanese Society of Gerodontology, and the Japanese Association on Sarcopenia and Frailty.” Geriatrics & Gerontology International 24, no. 11: 1111–1119. 10.1111/ggi.14980. 39375858 PMC11843523

[fsn370607-bib-0043] The Lancet . 2019. “Bringing Frailty Into All Realms of Medicine.” Lancet 394, no. 10206: 1298. 10.1016/s0140-6736(19)32279-2. 31609212

[fsn370607-bib-0044] Walker, K. A. , N. Basisty , D. M. Wilson 3rd , and L. Ferrucci . 2022. “Connecting Aging Biology and Inflammation in the Omics Era.” Journal of Clinical Investigation 132, no. 14: e158448. 10.1172/jci158448. 35838044 PMC9282936

[fsn370607-bib-0045] Wang, Y. , C. N. Uffelman , R. E. Bergia , et al. 2023. “Meat Consumption and Gut Microbiota: A Scoping Review of Literature and Systematic Review of Randomized Controlled Trials in Adults.” Advances in Nutrition 14, no. 2: 215–237. 10.1016/j.advnut.2022.10.005. 36822879 PMC10229385

[fsn370607-bib-0046] Wennberg, A. , Y. Tao , S. Ek , and K. Modig . 2023. “Population Frailty Trends by Education and Income Levels Over a Period of 30 Years: Findings From Swedish Registry Data.” Journal of Epidemiology and Community Health 78, no. 2: 109–114. 10.1136/jech-2023-221060. 37788900 PMC10850722

[fsn370607-bib-0047] Wilson, R. , J. Willis , R. Gearry , et al. 2017. “Inadequate Vitamin C Status in Prediabetes and Type 2 Diabetes Mellitus: Associations With Glycaemic Control, Obesity, and Smoking.” Nutrients 9, no. 9: 997. 10.3390/nu9090997. 28891932 PMC5622757

[fsn370607-bib-0048] Xu, Y. , Y. Wang , H. Li , et al. 2021. “Altered Fecal Microbiota Composition in Older Adults With Frailty.” Frontiers in Cellular and Infection Microbiology 11: 696186. 10.3389/fcimb.2021.696186. 34485176 PMC8415883

[fsn370607-bib-0049] Yoshihara, A. , K. Nakashima , K. Suwama , A. Odajima , T. Yamaga , and H. Ogawa . 2022. “Interaction Between Serum Vitamin C Levels and Smoking on the Periodontal Condition in Older Adults.” Journal of Periodontal Research 57, no. 3: 587–593. 10.1111/jre.12988. 35415888

[fsn370607-bib-0050] Yu, D. , S. M. Nguyen , Y. Yang , et al. 2021. “Long‐Term Diet Quality Is Associated With Gut Microbiome Diversity and Composition Among Urban Chinese Adults.” American Journal of Clinical Nutrition 113, no. 3: 684–694. 10.1093/ajcn/nqaa350. 33471054 PMC7948864

