# Supplementary material for: Association Between the Dietary Index for Gut Microbiota and Frailty: Mediating Role of Body Mass Index
Source: Food Sci Nutr. 2025 Jul 14;13(7):e70607. doi: 10.1002/fsn3.70607 (PMC12257151; doi:10.1002/fsn3.70607)
Supplement: Supplementary file 1 — Data S1. [file FSN3-13-e70607-s001.docx]

#
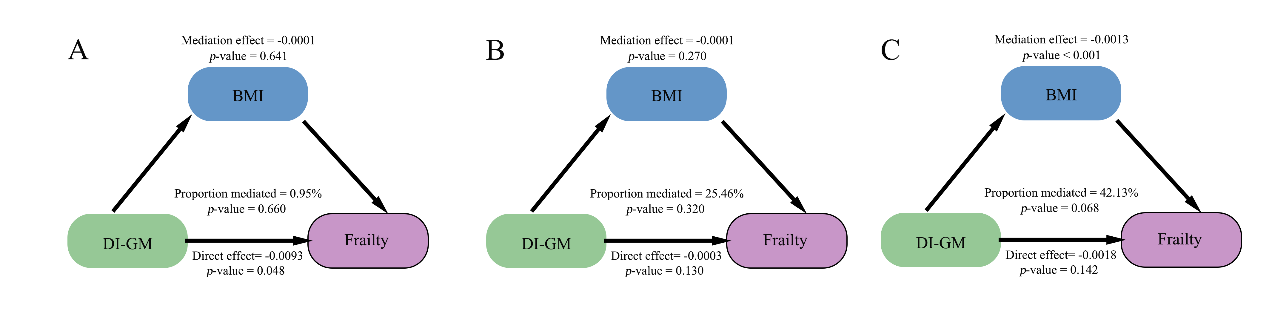


Figure S1: Stratified mediation analysis of BMI categories in the DI-GM-frailty association (A, <25 kg/m²; B, 25-30 kg/m²; C, >30 kg/m²).

# Table S1: Variables in Frailty Index and Respective Scores

| **Items** | | **scores** |
| --- | --- | --- |
| **Cognition** | |  |
| 1.experience confusion/memory problems | | yes=1, no=0 |
| **Dependence** | |  |
| 2.managing money difficulty | | no difficulty=0, Some difficulty=0.33, much difficulty=0.66, unable to do=1 |
| 3.walking for a quarter mile difficulty | | no difficulty=0, Some difficulty=0.33, much difficulty=0.66, unable to do=1 |
| 4.walking up ten steps difficulty | | no difficulty=0, Some difficulty=0.33, much difficulty=0.66, unable to do=1 |
| 5.stooping, crouching, kneeling difficulty | | no difficulty=0, Some difficulty=0.33, much difficulty=0.66, unable to do=1 |
| 6.lifting or carrying difficulty | | no difficulty=0, Some difficulty=0.33, much difficulty=0.66, unable to do=1 |
| 7.house chore difficulty | | no difficulty=0, Some difficulty=0.33, much difficulty=0.66, unable to do=1 |
| 8.preparing meals difficulty | | no difficulty=0, Some difficulty=0.33, much difficulty=0.66, unable to do=1 |
| 9.standing up from armless chair difficulty | | no difficulty=0, Some difficulty=0.33, much difficulty=0.66, unable to do=1 |
| 10.getting in and out of bed difficulty | | no difficulty=0, Some difficulty=0.33, much difficulty=0.66, unable to do=1 |
| 11.using fork, knife, drinking from cup difficulty | | no difficulty=0, Some difficulty=0.33, much difficulty=0.66, unable to do=1 |
| 12.dressing yourself difficulty | | no difficulty=0, Some difficulty=0.33, much difficulty=0.66, unable to do=1 |
| 13.standing for long periods difficulty | | no difficulty=0, Some difficulty=0.33, much difficulty=0.66, unable to do=1 |
| 14.grasp/holding small objects difficulty | | no difficulty=0, Some difficulty=0.33, much difficulty=0.66, unable to do=1 |
| 15.attending social event difficulty | | no difficulty=0, Some difficulty=0.33, much difficulty=0.66, unable to do=1 |
| 16.leisure activity at home difficulty | | no difficulty=0, Some difficulty=0.33, much difficulty=0.66, unable to do=1 |
| 17.push or pull large objects difficulty | | no difficulty=0, Some difficulty=0.33, much difficulty=0.66, unable to do=1 |
| **Depressive Conditions** | |  |
| 18.have little interest in doing things | nearly every day = 1, more than half the days = 0.66, several days = 0.33, no =0 | |
| 19.feeling down, depressed, or hopeless | nearly every day = 1, more than half the days = 0.66, several days = 0.33, no =0 | |
| 20.trouble sleeping or sleeping too much | nearly every day = 1, more than half the days = 0.66, several days = 0.33, no =0 | |
| 21.feeling tired or having little energy | nearly every day = 1, more than half the days = 0.66, several days = 0.33, no =0 | |
| 22.poor appetite or overeating | nearly every day = 1, more than half the days = 0.66, several days = 0.33, no =0 | |
| 23.feeling bad about yourself | nearly every day = 1, more than half the days = 0.66, several days = 0.33, no =0 | |
| 24.trouble concentrating on things | nearly every day = 1, more than half the days = 0.66, several days = 0.33, no =0 | |
| **Comorbidities** | |  |
| 25.doctor ever said you had arthritis | | yes = 1, no = 0 |
| 26.ever told you had thyroid problem | | yes = 1, no = 0 |
| 27.ever told you had chronic bronchitis | | yes = 1, no = 0 |
| 28.ever told you had cancer or malignancy | | yes = 1, no = 0 |
| 29.ever told had congestive heart failure | | yes = 1, no = 0 |
| 30.ever told you had coronary heart disease | | yes = 1, no = 0 |
| 31.ever told you had angina/angina pectoris | | yes = 1, no = 0 |
| 32.ever told you had heart attack | | yes = 1, no = 0 |
| 33.ever told you had a stroke | | yes = 1, no = 0 |
| 34.ever told you had high blood pressure | | yes = 1, no = 0 |
| 35.doctor told you have diabetes | | yes = 1, borderline=0.5, no =0 |
| 36.ever told you had weak/failing kidneys | | yes = 1, no =0 |
| 37.urine leakage bother you? | | greatly = 1, very much =0.75, somewhat= 0.5, only a little = 0.25, no=0 |
| **Hospital and Care** | |  |
| 38.general health condition | | excellent, very good, good = 0, fair, poor = 1 |
| 39.health now compared with 1 year ago | | worse =1, better = 0 |
| 40.overnight hospital patient in last year | | yes =1, no =0 |
| 41.times receive healthcare over past year | | no=0, 1-4=0.5, ≥5 =1 |
| 42.number of prescription medicines taken | | no =0, 1-4=0.5, ≥5 =1 |
| **Physical Anthropometry** | |  |
| 43.body mass index (kg/m^2^) | | <18.5, ≥30=1  ≥25, <30=0.5  ≥18.5,＜25=0 |
| **Laboratory values** | |  |
| 44.glycohemoglobin (%) | | 0%-5.7%=0, >5.7%=1 |
| 45.red blood cell count (million cells/ul) | | M: ≥4.7, <6.1=0, Other=1  F: ≥4.2,＜5.4=0, Other =1 |
| 46.hemoglobin (g/dl) | | M: ≥13.5, <18 =0, Other =1  F: ≥12, <16 =0, Other=1 |
| 47.red cell distribution width (%) | | ≥11.6, <14.6=0, Other=1 |
| 48.lymphocyte percent (%) | | ≥20, <40=0, Other=1 |
| 49.segmented neutrophils percent (%) | | ≥40, <80=0, Other=1 |
